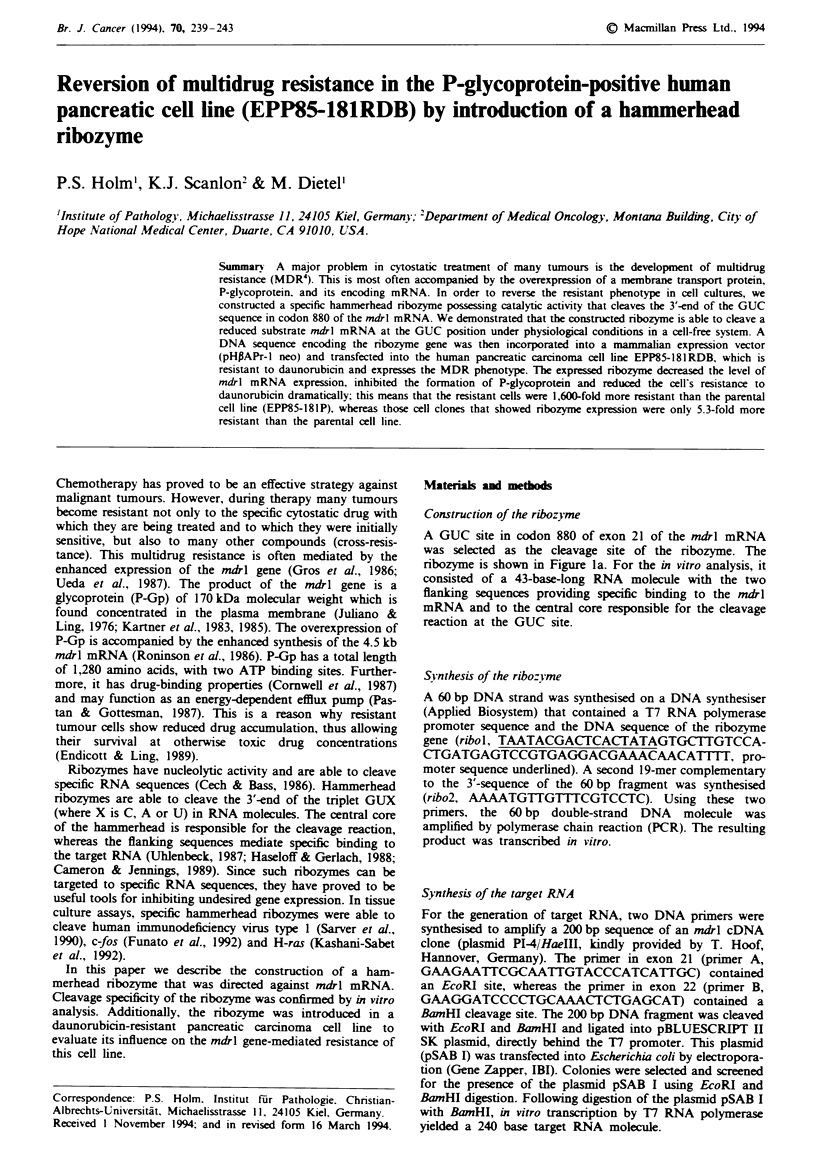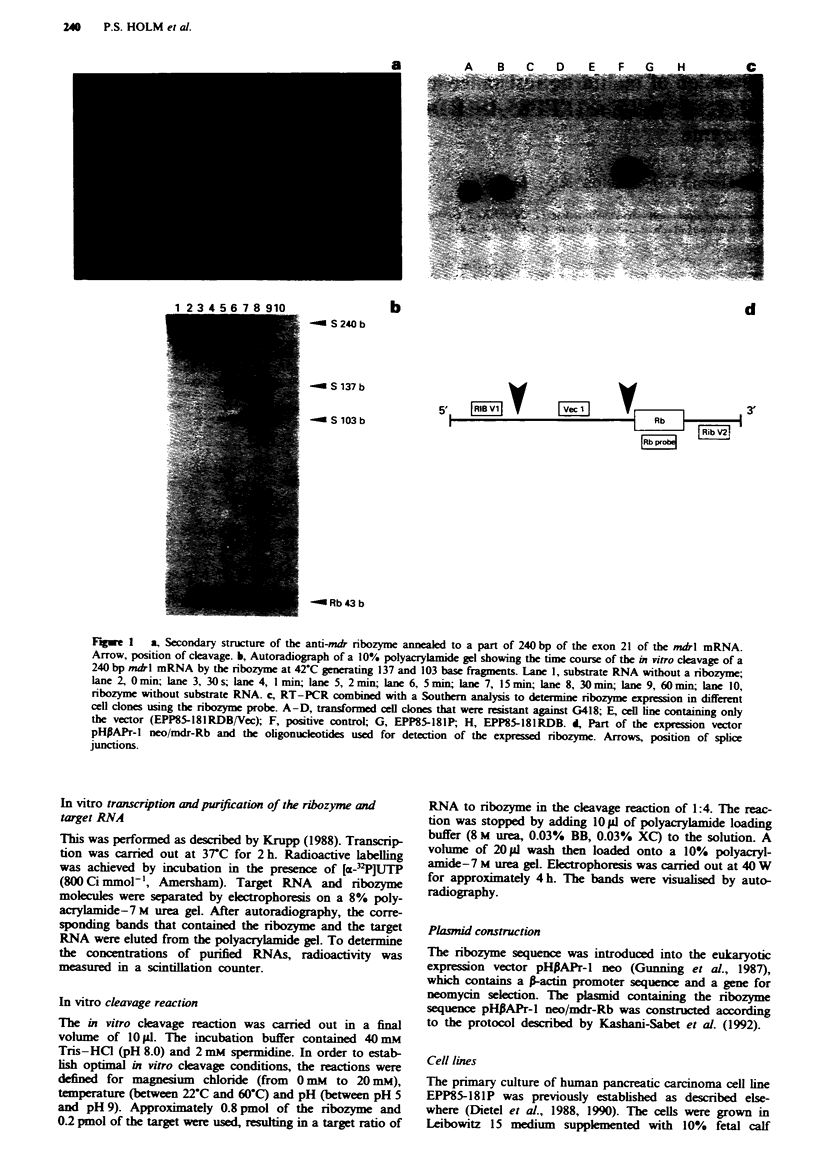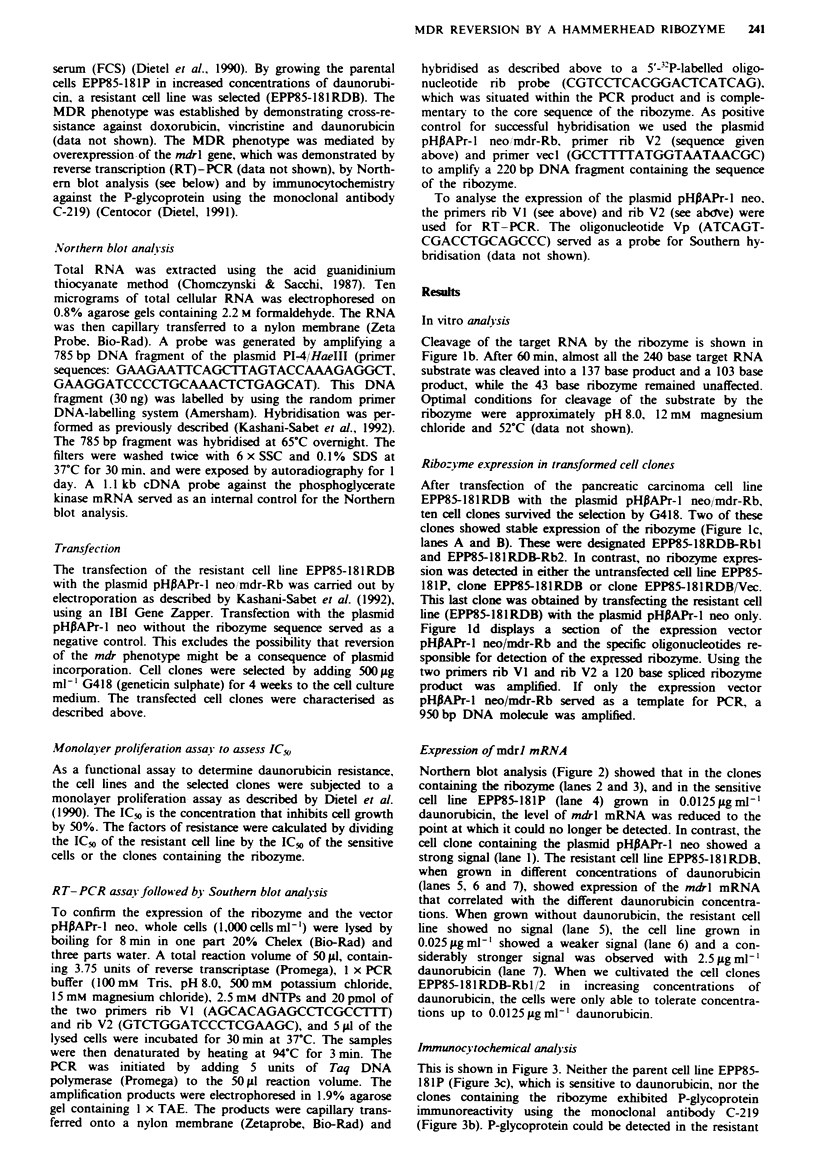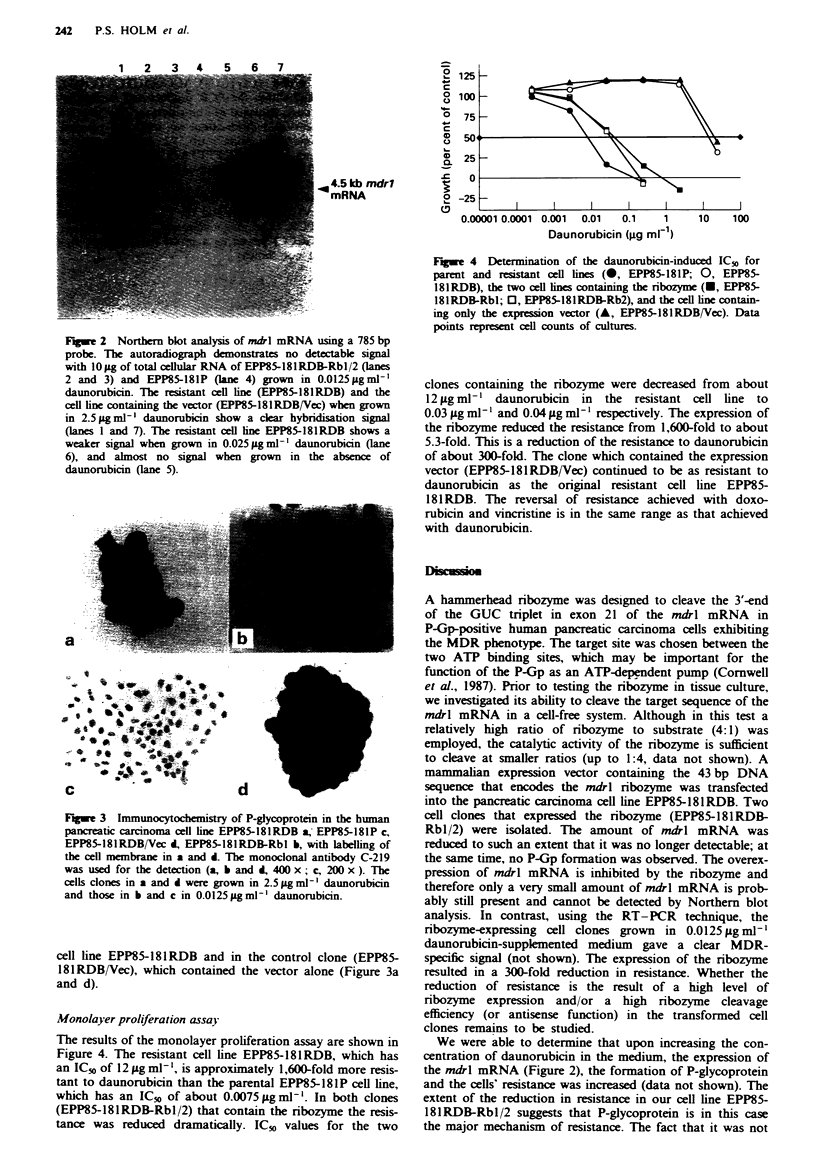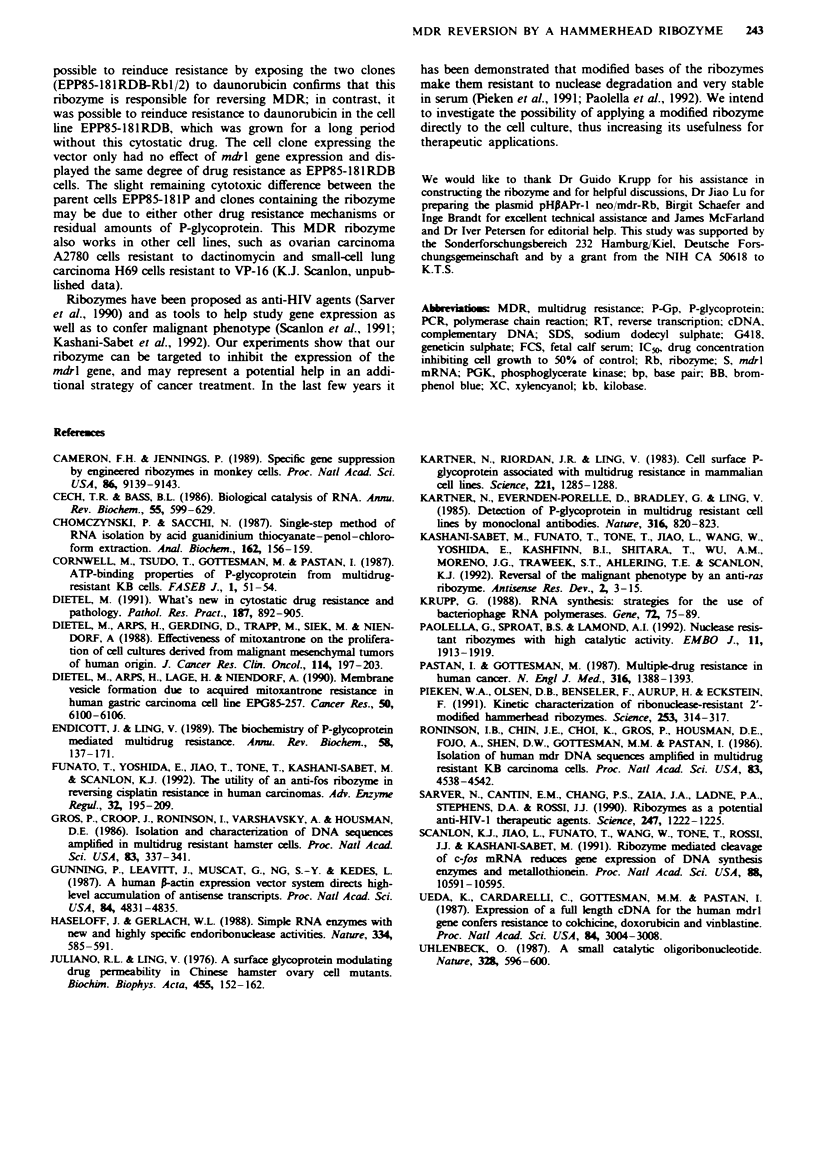# Reversion of multidrug resistance in the P-glycoprotein-positive human pancreatic cell line (EPP85-181RDB) by introduction of a hammerhead ribozyme.

**DOI:** 10.1038/bjc.1994.286

**Published:** 1994-08

**Authors:** P. S. Holm, K. J. Scanlon, M. Dietel

**Affiliations:** Institute of Pathology, Kiel, Germany.

## Abstract

**Images:**


Br. J. Cancer (1994), 70, 239-243 © Macmillan Press Ltd., 1994

### Reversion of multidrug resistance in the P-glycoprotein-positive human pancreatic cell line (EPP85-181RDB) by introduction of a hammerhead ribozyme

P.S. Holm[1], K.J. Scanlon[2] & M. Dietel[1]

[1]*Institute of Pathology, Michaelisstrasse 11, 24105 Kiel, Germany;* [2]*Department of Medical Oncology, Montana Building, City of Hope National Medical Center, Duarte, CA 91010, USA.*

**Summary** A major problem in cytostatic treatment of many tumours is the development of multidrug resistance (MDR[4]). This is most often accompanied by the overexpression of a membrane transport protein, P-glycoprotein, and its encoding mRNA. In order to reverse the resistant phenotype in cell cultures, we constructed a specific hammerhead ribozyme possessing catalytic activity that cleaves the 3′-end of the GUC sequence in codon 880 of the *mdr*1 mRNA. We demonstrated that the constructed ribozyme is able to cleave a reduced substrate *mdr*1 mRNA at the GUC position under physiological conditions in a cell-free system. A DNA sequence encoding the ribozyme gene was then incorporated into a mammalian expression vector (pHβAPr-1 neo) and transfected into the human pancreatic carcinoma cell line EPP85-181RDB, which is resistant to daunorubicin and expresses the MDR phenotype. The expressed ribozyme decreased the level of *mdr*1 mRNA expression, inhibited the formation of P-glycoprotein and reduced the cell's resistance to daunorubicin dramatically; this means that the resistant cells were 1,600-fold more resistant than the parental cell line (EPP85-181P), whereas those cell clones that showed ribozyme expression were only 5.3-fold more resistant than the parental cell line.

Chemotherapy has proved to be an effective strategy against malignant tumours. However, during therapy many tumours become resistant not only to the specific cytostatic drug with which they are being treated and to which they were initially sensitive, but also to many other compounds (cross-resistance). This multidrug resistance is often mediated by the enhanced expression of the *mdr*1 gene (Gros *et al.*, 1986; Ueda *et al.*, 1987). The product of the *mdr*1 gene is a glycoprotein (P-Gp) of 170 kDa molecular weight which is found concentrated in the plasma membrane (Juliano & Ling, 1976; Kartner *et al.*, 1983, 1985). The overexpression of P-Gp is accompanied by the enhanced synthesis of the 4.5 kb *mdr*1 mRNA (Roninson *et al.*, 1986). P-Gp has a total length of 1,280 amino acids, with two ATP binding sites. Furthermore, it has drug-binding properties (Cornwell *et al.*, 1987) and may function as an energy-dependent efflux pump (Pastan & Gottesman, 1987). This is a reason why resistant tumour cells show reduced drug accumulation, thus allowing their survival at otherwise toxic drug concentrations (Endicott & Ling, 1989).

Ribozymes have nucleolytic activity and are able to cleave specific RNA sequences (Cech & Bass, 1986). Hammerhead ribozymes are able to cleave the 3′-end of the triplet GUX (where X is C, A or U) in RNA molecules. The central core of the hammerhead is responsible for the cleavage reaction, whereas the flanking sequences mediate specific binding to the target RNA (Uhlenbeck, 1987; Haseloff & Gerlach, 1988; Cameron & Jennings, 1989). Since such ribozymes can be targeted to specific RNA sequences, they have proved to be useful tools for inhibiting undesired gene expression. In tissue culture assays, specific hammerhead ribozymes were able to cleave human immunodeficiency virus type 1 (Sarver *et al.*, 1990), c-*fos* (Funato *et al.*, 1992) and H-*ras* (Kashani-Sabet *et al.*, 1992).

In this paper we describe the construction of a hammerhead ribozyme that was directed against *mdr*1 mRNA. Cleavage specificity of the ribozyme was confirmed by *in vitro* analysis. Additionally, the ribozyme was introduced in a daunorubicin-resistant pancreatic carcinoma cell line to evaluate its influence on the *mdr*1 gene-mediated resistance of this cell line.

Correspondence: P.S. Holm, Institut für Pathologie, Christian-Albrechts-Universität, Michaelisstrasse 11, 24105 Kiel, Germany.
Received 1 November 1994; and in revised form 16 March 1994.

### Materials and methods

### *Construction of the ribozyme*

A GUC site in codon 880 of exon 21 of the *mdr*1 mRNA was selected as the cleavage site of the ribozyme. The ribozyme is shown in Figure 1a. For the *in vitro* analysis, it consisted of a 43-base-long RNA molecule with the two flanking sequences providing specific binding to the *mdr*1 mRNA and to the central core responsible for the cleavage reaction at the GUC site.

### *Synthesis of the ribozyme*

A 60 bp DNA strand was synthesised on a DNA synthesiser (Applied Biosystem) that contained a T7 RNA polymerase promoter sequence and the DNA sequence of the ribozyme gene (*ribo*1, TAATACGACTCACTATAGTGCTTGTCCA-CTGATGAGTCCGTGAGGACGAAACAACATTTT, promoter sequence underlined). A second 19-mer complementary to the 3′-sequence of the 60 bp fragment was synthesised (*ribo*2, AAAATGTTGTTTCGTCCTC). Using these two primers, the 60 bp double-strand DNA molecule was amplified by polymerase chain reaction (PCR). The resulting product was transcribed *in vitro*.

### *Synthesis of the target RNA*

For the generation of target RNA, two DNA primers were synthesised to amplify a 200 bp sequence of an *mdr*1 cDNA clone (plasmid PI-4/*Hae*III, kindly provided by T. Hoof, Hannover, Germany). The primer in exon 21 (primer A, GAAGAATTCGCAATTGTACCCATCATTGC) contained an *Eco*RI site, whereas the primer in exon 22 (primer B, GAAGGATCCCCTGCAAACTCTGAGCAT) contained a *Bam*HI cleavage site. The 200 bp DNA fragment was cleaved with *Eco*RI and *Bam*HI and ligated into pBLUESCRIPT II SK plasmid, directly behind the T7 promoter. This plasmid (pSAB I) was transfected into *Escherichia coli* by electroporation (Gene Zapper, IBI). Colonies were selected and screened for the presence of the plasmid pSAB I using *Eco*RI and *Bam*HI digestion. Following digestion of the plasmid pSAB I with *Bam*HI, *in vitro* transcription by T7 RNA polymerase yielded a 240 base target RNA molecule.

**Figure 1** **a**, Secondary structure of the anti-*mdr* ribozyme annealed to a part of 240 bp of the exon 21 of the *mdr*1 mRNA. Arrow, position of cleavage. **b**, Autoradiograph of a 10% polyacrylamide gel showing the time course of the *in vitro* cleavage of a 240 bp *mdr*1 mRNA by the ribozyme at 42°C generating 137 and 103 base fragments. Lane 1, substrate RNA without a ribozyme; lane 2, 0 min; lane 3, 30 s; lane 4, 1 min; lane 5, 2 min; lane 6, 5 min; lane 7, 15 min; lane 8, 30 min; lane 9, 60 min; lane 10, ribozyme without substrate RNA. **c**, RT-PCR combined with a Southern analysis to determine ribozyme expression in different cell clones using the ribozyme probe. A-D, transformed cell clones that were resistant against G418; E, cell line containing only the vector (EPP85-181RDB/Vec); F, positive control; G, EPP85-181P; H, EPP85-181RDB. **d**, Part of the expression vector pH$\beta$APr-1 neo/mdr-Rb and the oligonucleotides used for detection of the expressed ribozyme. Arrows, position of splice junctions.

### In vitro *transcription and purification of the ribozyme and target RNA*

This was performed as described by Krupp (1988). Transcription was carried out at 37°C for 2 h. Radioactive labelling was achieved by incubation in the presence of [$\alpha$-$^{32}$P]UTP (800 Ci mmol$^{-1}$, Amersham). Target RNA and ribozyme molecules were separated by electrophoresis on a 8% polyacrylamide-7 M urea gel. After autoradiography, the corresponding bands that contained the ribozyme and the target RNA were eluted from the polyacrylamide gel. To determine the concentrations of purified RNAs, radioactivity was measured in a scintillation counter.

### In vitro *cleavage reaction*

The *in vitro* cleavage reaction was carried out in a final volume of 10 µl. The incubation buffer contained 40 mM Tris-HCl (pH 8.0) and 2 mM spermidine. In order to establish optimal *in vitro* cleavage conditions, the reactions were defined for magnesium chloride (from 0 mM to 20 mM), temperature (between 22°C and 60°C) and pH (between pH 5 and pH 9). Approximately 0.8 pmol of the ribozyme and 0.2 pmol of the target were used, resulting in a target ratio of RNA to ribozyme in the cleavage reaction of 1:4. The reaction was stopped by adding 10 µl of polyacrylamide loading buffer (8 M urea, 0.03% BB, 0.03% XC) to the solution. A volume of 20 µl wash then loaded onto a 10% polyacrylamide-7 M urea gel. Electrophoresis was carried out at 40 W for approximately 4 h. The bands were visualised by autoradiography.

### *Plasmid construction*

The ribozyme sequence was introduced into the eukaryotic expression vector pH$\beta$APr-1 neo (Gunning *et al.*, 1987), which contains a $\beta$-actin promoter sequence and a gene for neomycin selection. The plasmid containing the ribozyme sequence pH$\beta$APr-1 neo/mdr-Rb was constructed according to the protocol described by Kashani-Sabet *et al.* (1992).

### *Cell lines*

The primary culture of human pancreatic carcinoma cell line EPP85-181P was previously established as described elsewhere (Dietel *et al.*, 1988, 1990). The cells were grown in Leibowitz 15 medium supplemented with 10% fetal calf serum (FCS) (Dietel *et al.*, 1990). By growing the parental cells EPP85-181P in increased concentrations of daunorubicin, a resistant cell line was selected (EPP85-181RDB). The MDR phenotype was established by demonstrating cross-resistance against doxorubicin, vincristine and daunorubicin (data not shown). The MDR phenotype was mediated by overexpression of the *mdr*1 gene, which was demonstrated by reverse transcription (RT)-PCR (data not shown), by Northern blot analysis (see below) and by immunocytochemistry against the P-glycoprotein using the monoclonal antibody C-219) (Centocor (Dietel, 1991).

### *Northern blot analysis*

Total RNA was extracted using the acid guanidinium thiocyanate method (Chomczynski & Sacchi, 1987). Ten micrograms of total cellular RNA was electrophoresed on 0.8% agarose gels containing 2.2 M formaldehyde. The RNA was then capillary transferred to a nylon membrane (Zeta Probe, Bio-Rad). A probe was generated by amplifying a 785 bp DNA fragment of the plasmid PI-4/*Hae*III (primer sequences: GAAGAATTCAGCTTAGTACCAAAGAGGCT, GAAGGATCCCCTGCAAACTCTGAGCAT). This DNA fragment (30 ng) was labelled by using the random primer DNA-labelling system (Amersham). Hybridisation was performed as previously described (Kashani-Sabet *et al.*, 1992). The 785 bp fragment was hybridised at 65°C overnight. The filters were washed twice with 6 × SSC and 0.1% SDS at 37°C for 30 min, and were exposed by autoradiography for 1 day. A 1.1 kb cDNA probe against the phosphoglycerate kinase mRNA served as an internal control for the Northern blot analysis.

### *Transfection*

The transfection of the resistant cell line EPP85-181RDB with the plasmid pH$\beta$APr-1 neo/mdr-Rb was carried out by electroporation as described by Kashani-Sabet *et al.* (1992), using an IBI Gene Zapper. Transfection with the plasmid pH$\beta$APr-1 neo without the ribozyme sequence served as a negative control. This excludes the possibility that reversion of the *mdr* phenotype might be a consequence of plasmid incorporation. Cell clones were selected by adding 500 $\mu$g ml$^{-1}$ G418 (geneticin sulphate) for 4 weeks to the cell culture medium. The transfected cell clones were characterised as described above.

### *Monolayer proliferation assay to assess $IC_{50}$*

As a functional assay to determine daunorubicin resistance, the cell lines and the selected clones were subjected to a monolayer proliferation assay as described by Dietel *et al.* (1990). The $IC_{50}$ is the concentration that inhibits cell growth by 50%. The factors of resistance were calculated by dividing the $IC_{50}$ of the resistant cell line by the $IC_{50}$ of the sensitive cells or the clones containing the ribozyme.

### *RT-PCR assay followed by Southern blot analysis*

To confirm the expression of the ribozyme and the vector pH$\beta$APr-1 neo, whole cells (1,000 cells ml$^{-1}$) were lysed by boiling for 8 min in one part 20% Chelex (Bio-Rad) and three parts water. A total reaction volume of 50 $\mu$l, containing 3.75 units of reverse transcriptase (Promega), 1 × PCR buffer (100 mM Tris, pH 8.0, 500 mM potassium chloride, 15 mM magnesium chloride), 2.5 mM dNTPs and 20 pmol of the two primers rib V1 (AGCACAGAGCCTCGCCTTT) and rib V2 (GTCTGGATCCCTCGAAGC), and 5 $\mu$l of the lysed cells were incubated for 30 min at 37°C. The samples were then denaturated by heating at 94°C for 3 min. The PCR was initiated by adding 5 units of *Taq* DNA polymerase (Promega) to the 50 $\mu$l reaction volume. The amplification products were electrophoresed in 1.9% agarose gel containing 1 × TAE. The products were capillary transferred onto a nylon membrane (Zetaprobe, Bio-Rad) and hybridised as described above to a 5'-$^{32}$P-labelled oligonucleotide rib probe (CGTCCTCACGGACTCATCAG), which was situated within the PCR product and is complementary to the core sequence of the ribozyme. As positive control for successful hybridisation we used the plasmid pH$\beta$APr-1 neo/mdr-Rb, primer rib V2 (sequence given above) and primer vec1 (GCCTTTTATGGTAATAACGC) to amplify a 220 bp DNA fragment containing the sequence of the ribozyme.

To analyse the expression of the plasmid pH$\beta$APr-1 neo, the primers rib V1 (see above) and rib V2 (see above) were used for RT-PCR. The oligonucleotide Vp (ATCAGT-CGACCTGCAGCCC) served as a probe for Southern hybridisation (data not shown).

### Results

### In vitro *analysis*

Cleavage of the target RNA by the ribozyme is shown in Figure 1b. After 60 min, almost all the 240 base target RNA substrate was cleaved into a 137 base product and a 103 base product, while the 43 base ribozyme remained unaffected. Optimal conditions for cleavage of the substrate by the ribozyme were approximately pH 8.0, 12 mM magnesium chloride and 52°C (data not shown).

### *Ribozyme expression in transformed cell clones*

After transfection of the pancreatic carcinoma cell line EPP85-181RDB with the plasmid pH$\beta$APr-1 neo/mdr-Rb, ten cell clones survived the selection by G418. Two of these clones showed stable expression of the ribozyme (Figure 1c, lanes A and B). These were designated EPP85-18RDB-Rb1 and EPP85-181RDB-Rb2. In contrast, no ribozyme expression was detected in either the untransfected cell line EPP85-181P, clone EPP85-181RDB or clone EPP85-181RDB/Vec. This last clone was obtained by transfecting the resistant cell line (EPP85-181RDB) with the plasmid pH$\beta$APr-1 neo only. Figure 1d displays a section of the expression vector pH$\beta$APr-1 neo/mdr-Rb and the specific oligonucleotides responsible for detection of the expressed ribozyme. Using the two primers rib V1 and rib V2 a 120 base spliced ribozyme product was amplified. If only the expression vector pH$\beta$APr-1 neo/mdr-Rb served as a template for PCR, a 950 bp DNA molecule was amplified.

### *Expression of* mdr1 *mRNA*

Northern blot analysis (Figure 2) showed that in the clones containing the ribozyme (lanes 2 and 3), and in the sensitive cell line EPP85-181P (lane 4) grown in 0.0125 $\mu$g ml$^{-1}$ daunorubicin, the level of *mdr*1 mRNA was reduced to the point at which it could no longer be detected. In contrast, the cell clone containing the plasmid pH$\beta$APr-1 neo showed a strong signal (lane 1). The resistant cell line EPP85-181RDB, when grown in different concentrations of daunorubicin (lanes 5, 6 and 7), showed expression of the *mdr*1 mRNA that correlated with the different daunorubicin concentrations. When grown without daunorubicin, the resistant cell line showed no signal (lane 5), the cell line grown in 0.025 $\mu$g ml$^{-1}$ showed a weaker signal (lane 6) and a considerably stronger signal was observed with 2.5 $\mu$g ml$^{-1}$ daunorubicin (lane 7). When we cultivated the cell clones EPP85-181RDB-Rb1/2 in increasing concentrations of daunorubicin, the cells were only able to tolerate concentrations up to 0.0125 $\mu$g ml$^{-1}$ daunorubicin.

### *Immunocytochemical analysis*

This is shown in Figure 3. Neither the parent cell line EPP85-181P (Figure 3c), which is sensitive to daunorubicin, nor the clones containing the ribozyme exhibited P-glycoprotein immunoreactivity using the monoclonal antibody C-219 (Figure 3b). P-glycoprotein could be detected in the resistant

**Figure 2** Northern blot analysis of *mdr*1 mRNA using a 785 bp probe. The autoradiograph demonstrates no detectable signal with 10 μg of total cellular RNA of EPP85-181RDB-Rb1/2 (lanes 2 and 3) and EPP85-181P (lane 4) grown in 0.0125 μg ml$^{-1}$ daunorubicin. The resistant cell line (EPP85-181RDB) and the cell line containing the vector (EPP85-181RDB/Vec) when grown in 2.5 μg ml$^{-1}$ daunorubicin show a clear hybridisation signal (lanes 1 and 7). The resistant cell line EPP85-181RDB shows a weaker signal when grown in 0.025 μg ml$^{-1}$ daunorubicin (lane 6), and almost no signal when grown in the absence of daunorubicin (lane 5).

**Figure 3** Immunocytochemistry of P-glycoprotein in the human pancreatic carcinoma cell line EPP85-181RDB **a**, EPP85-181P **c**, EPP85-181RDB/Vec **d**, EPP85-181RDB-Rb1 **b**, with labelling of the cell membrane in **a** and **d**. The monoclonal antibody C-219 was used for the detection (**a**, **b** and **d**, 400 × ; **c**, 200 × ). The cells clones in **a** and **d** were grown in 2.5 μg ml$^{-1}$ daunorubicin and those in **b** and **c** in 0.0125 μg ml$^{-1}$ daunorubicin.

cell line EPP85-181RDB and in the control clone (EPP85-181RDB/Vec), which contained the vector alone (Figure 3a and d).

*Monolayer proliferation assay*

The results of the monolayer proliferation assay are shown in Figure 4. The resistant cell line EPP85-181RDB, which has an $IC_{50}$ of 12 μg ml$^{-1}$, is approximately 1,600-fold more resistant to daunorubicin than the parental EPP85-181P cell line, which has an $IC_{50}$ of about 0.0075 μg ml$^{-1}$. In both clones (EPP85-181RDB-Rb1/2) that contain the ribozyme the resistance was reduced dramatically. $IC_{50}$ values for the two

**Figure 4** Determination of the daunorubicin-induced $IC_{50}$ for parent and resistant cell lines (●, EPP85-181P; ○, EPP85-181RDB), the two cell lines containing the ribozyme (■, EPP85-181RDB-Rb1; □, EPP85-181RDB-Rb2), and the cell line containing only the expression vector (▲, EPP85-181RDB/Vec). Data points represent cell counts of cultures.

clones containing the ribozyme were decreased from about 12 μg ml$^{-1}$ daunorubicin in the resistant cell line to 0.03 μg ml$^{-1}$ and 0.04 μg ml$^{-1}$ respectively. The expression of the ribozyme reduced the resistance from 1,600-fold to about 5.3-fold. This is a reduction of the resistance to daunorubicin of about 300-fold. The clone which contained the expression vector (EPP85-181RDB/Vec) continued to be as resistant to daunorubicin as the original resistant cell line EPP85-181RDB. The reversal of resistance achieved with doxorubicin and vincristine is in the same range as that achieved with daunorubicin.

### Discussion

A hammerhead ribozyme was designed to cleave the 3′-end of the GUC triplet in exon 21 of the *mdr*1 mRNA in P-Gp-positive human pancreatic carcinoma cells exhibiting the MDR phenotype. The target site was chosen between the two ATP binding sites, which may be important for the function of the P-Gp as an ATP-dependent pump (Cornwell *et al*., 1987). Prior to testing the ribozyme in tissue culture, we investigated its ability to cleave the target sequence of the *mdr*1 mRNA in a cell-free system. Although in this test a relatively high ratio of ribozyme to substrate (4:1) was employed, the catalytic activity of the ribozyme is sufficient to cleave at smaller ratios (up to 1:4, data not shown). A mammalian expression vector containing the 43 bp DNA sequence that encodes the *mdr*1 ribozyme was transfected into the pancreatic carcinoma cell line EPP85-181RDB. Two cell clones that expressed the ribozyme (EPP85-181RDB-Rb1/2) were isolated. The amount of *mdr*1 mRNA was reduced to such an extent that it was no longer detectable; at the same time, no P-Gp formation was observed. The overexpression of *mdr*1 mRNA is inhibited by the ribozyme and therefore only a very small amount of *mdr*1 mRNA is probably still present and cannot be detected by Northern blot analysis. In contrast, using the RT–PCR technique, the ribozyme-expressing cell clones grown in 0.0125 μg ml$^{-1}$ daunorubicin-supplemented medium gave a clear MDR-specific signal (not shown). The expression of the ribozyme resulted in a 300-fold reduction in resistance. Whether the reduction of resistance is the result of a high level of ribozyme expression and/or a high ribozyme cleavage efficiency (or antisense function) in the transformed cell clones remains to be studied.

We were able to determine that upon increasing the concentration of daunorubicin in the medium, the expression of the *mdr*1 mRNA (Figure 2), the formation of P-glycoprotein and the cells' resistance was increased (data not shown). The extent of the reduction in resistance in our cell line EPP85-181RDB-Rb1/2 suggests that P-glycoprotein is in this case the major mechanism of resistance. The fact that it was not possible to reinduce resistance by exposing the two clones (EPP85-181RDB-Rb1/2) to daunorubicin confirms that this ribozyme is responsible for reversing MDR; in contrast, it was possible to reinduce resistance to daunorubicin in the cell line EPP85-181RDB, which was grown for a long period without this cytostatic drug. The cell clone expressing the vector only had no effect of *mdr*1 gene expression and displayed the same degree of drug resistance as EPP85-181RDB cells. The slight remaining cytotoxic difference between the parent cells EPP85-181P and clones containing the ribozyme may be due to either other drug resistance mechanisms or residual amounts of P-glycoprotein. This MDR ribozyme also works in other cell lines, such as ovarian carcinoma A2780 cells resistant to dactinomycin and small-cell lung carcinoma H69 cells resistant to VP-16 (K.J. Scanlon, unpublished data).

Ribozymes have been proposed as anti-HIV agents (Sarver *et al.*, 1990) and as tools to help study gene expression as well as to confer malignant phenotype (Scanlon *et al.*, 1991; Kashani-Sabet *et al.*, 1992). Our experiments show that our ribozyme can be targeted to inhibit the expression of the *mdr*1 gene, and may represent a potential help in an additional strategy of cancer treatment. In the last few years it has been demonstrated that modified bases of the ribozymes make them resistant to nuclease degradation and very stable in serum (Pieken *et al.*, 1991; Paolella *et al.*, 1992). We intend to investigate the possibility of applying a modified ribozyme directly to the cell culture, thus increasing its usefulness for therapeutic applications.

We would like to thank Dr Guido Krupp for his assistance in constructing the ribozyme and for helpful discussions, Dr Jiao Lu for preparing the plasmid pH$\beta$APr-1 neo/mdr-Rb, Birgit Schaefer and Inge Brandt for excellent technical assistance and James McFarland and Dr Iver Petersen for editorial help. This study was supported by the Sonderforschungsbereich 232 Hamburg/Kiel, Deutsche Forschungsgemeinschaft and by a grant from the NIH CA 50618 to K.T.S.

**Abbreviations:** MDR, multidrug resistance; P-Gp, P-glycoprotein; PCR, polymerase chain reaction; RT, reverse transcription; cDNA, complementary DNA; SDS, sodium dodecyl sulphate; G418, geneticin sulphate; FCS, fetal calf serum; $IC_{50}$, drug concentration inhibiting cell growth to 50% of control; Rb, ribozyme; S, *mdr*1 mRNA; PGK, phosphoglycerate kinase; bp, base pair; BB, bromphenol blue; XC, xylencyanol; kb, kilobase.

### References

CAMERON, F.H. & JENNINGS, P. (1989). Specific gene suppression by engineered ribozymes in monkey cells. *Proc. Natl Acad. Sci. USA*, **86**, 9139–9143.

CECH, T.R. & BASS, B.L. (1986). Biological catalysis of RNA. *Annu. Rev. Biochem.*, **55**, 599–629.

CHOMCZYNSKI, P. & SACCHI, N. (1987). Single-step method of RNA isolation by acid guanidinium thiocyanate–penol–chloroform extraction. *Anal. Biochem.*, **162**, 156–159.

CORNWELL, M., TSUDO, T., GOTTESMAN, M. & PASTAN, I. (1987). ATP-binding properties of P-glycoprotein from multidrug-resistant KB cells. *FASEB J.*, **1**, 51–54.

DIETEL, M. (1991). What's new in cytostatic drug resistance and pathology. *Pathol. Res. Pract.*, **187**, 892–905.

DIETEL, M., ARPS, H., GERDING, D., TRAPP, M., SIEK, M. & NIENDORF, A (1988). Effectiveness of mitoxantrone on the proliferation of cell cultures derived from malignant mesenchymal tumors of human origin. *J. Cancer Res. Clin. Oncol.*, **114**, 197–203.

DIETEL, M., ARPS, H., LAGE, H. & NIENDORF, A. (1990). Membrane vesicle formation due to acquired mitoxantrone resistance in human gastric carcinoma cell line EPG85-257. *Cancer Res.*, **50**, 6100–6106.

ENDICOTT, J. & LING, V. (1989). The biochemistry of P-glycoprotein mediated multidrug resistance. *Annu. Rev. Biochem.*, **58**, 137–171.

FUNATO, T., YOSHIDA, E., JIAO, T., TONE, T., KASHANI-SABET, M. & SCANLON, K.J. (1992). The utility of an anti-fos ribozyme in reversing cisplatin resistance in human carcinomas. *Adv. Enzyme Regul.*, **32**, 195–209.

GROS, P., CROOP, J., RONINSON, I., VARSHAVSKY, A. & HOUSMAN, D.E. (1986). Isolation and characterization of DNA sequences amplified in multidrug resistant hamster cells. *Proc. Natl Acad. Sci. USA*, **83**, 337–341.

GUNNING, P., LEAVITT, J., MUSCAT, G., NG, S.-Y. & KEDES, L. (1987). A human $\beta$-actin expression vector system directs high-level accumulation of antisense transcripts. *Proc. Natl Acad. Sci. USA*, **84**, 4831–4835.

HASELOFF, J. & GERLACH, W.L. (1988). Simple RNA enzymes with new and highly specific endoribonuclease activities. *Nature*, **334**, 585–591.

JULIANO, R.L. & LING, V. (1976). A surface glycoprotein modulating drug permeability in Chinese hamster ovary cell mutants. *Biochim. Biophys. Acta*, **455**, 152–162.

KARTNER, N., RIORDAN, J.R. & LING, V. (1983). Cell surface P-glycoprotein associated with multidrug resistance in mammalian cell lines. *Science*, **221**, 1285–1288.

KARTNER, N., EVERNDEN-PORELLE, D., BRADLEY, G. & LING, V. (1985). Detection of P-glycoprotein in multidrug resistant cell lines by monoclonal antibodies. *Nature*, **316**, 820–823.

KASHANI-SABET, M., FUNATO, T., TONE, T., JIAO, L., WANG, W., YOSHIDA, E., KASHFINN, B.I., SHITARA, T., WU, A.M., MORENO, J.G., TRAWEEK, S.T., AHLERING, T.E. & SCANLON, K.J. (1992). Reversal of the malignant phenotype by an anti-*ras* ribozyme. *Antisense Res. Dev.*, **2**, 3–15.

KRUPP, G. (1988). RNA synthesis: strategies for the use of bacteriophage RNA polymerases. *Gene*, **72**, 75–89.

PAOLELLA, G., SPROAT, B.S. & LAMOND, A.I. (1992). Nuclease resistant ribozymes with high catalytic activity. *EMBO J.*, **11**, 1913–1919.

PASTAN, I. & GOTTESMAN, M. (1987). Multiple-drug resistance in human cancer. *N. Engl J. Med.*, **316**, 1388–1393.

PIEKEN, W.A., OLSEN, D.B., BENSELER, F., AURUP, H. & ECKSTEIN, F. (1991). Kinetic characterization of ribonuclease-resistant 2′-modified hammerhead ribozymes. *Science*, **253**, 314–317.

RONINSON, I.B., CHIN, J.E., CHOI, K., GROS, P., HOUSMAN, D.E., FOJO, A., SHEN, D.W., GOTTESMAN, M.M. & PASTAN, I. (1986). Isolation of human mdr DNA sequences amplified in multidrug resistant KB carcinoma cells. *Proc. Natl Acad. Sci. USA*, **83**, 4538–4542.

SARVER, N., CANTIN, E.M., CHANG, P.S., ZAIA, J.A., LADNE, P.A., STEPHENS, D.A. & ROSSI, J.J. (1990). Ribozymes as a potential anti-HIV-1 therapeutic agents. *Science*, **247**, 1222–1225.

SCANLON, K.J., JIAO, L., FUNATO, T., WANG, W., TONE, T., ROSSI, J.J. & KASHANI-SABET, M. (1991). Ribozyme mediated cleavage of *c-fos* mRNA reduces gene expression of DNA synthesis enzymes and metallothionein. *Proc. Natl Acad. Sci. USA*, **88**, 10591–10595.

UEDA, K., CARDARELLI, C., GOTTESMAN, M.M. & PASTAN, I. (1987). Expression of a full length cDNA for the human mdr1 gene confers resistance to colchicine, doxorubicin and vinblastine. *Proc. Natl Acad. Sci. USA*, **84**, 3004–3008.

UHLENBECK, O. (1987). A small catalytic oligoribonucleotide. *Nature*, **328**, 596–600.